# Half-UNet: A Simplified U-Net Architecture for Medical Image Segmentation

**DOI:** 10.3389/fninf.2022.911679

**Published:** 2022-06-09

**Authors:** Haoran Lu, Yifei She, Jun Tie, Shengzhou Xu

**Affiliations:** ^1^College of Computer Science and Technology, South-Central Minzu University for Nationalities, Wuhan, China; ^2^Hubei Provincial Engineering Research Center for Intelligent Management of Manufacturing Enterprises, Wuhan, China

**Keywords:** medical image, segmentation, deep learning, U-Net, Half-UNet

## Abstract

Medical image segmentation plays a vital role in computer-aided diagnosis procedures. Recently, U-Net is widely used in medical image segmentation. Many variants of U-Net have been proposed, which attempt to improve the network performance while keeping the U-shaped structure unchanged. However, this U-shaped structure is not necessarily optimal. In this article, the effects of different parts of the U-Net on the segmentation ability are experimentally analyzed. Then a more efficient architecture, Half-UNet, is proposed. The proposed architecture is essentially an encoder-decoder network based on the U-Net structure, in which both the encoder and decoder are simplified. The re-designed architecture takes advantage of the unification of channel numbers, full-scale feature fusion, and Ghost modules. We compared Half-UNet with U-Net and its variants across multiple medical image segmentation tasks: mammography segmentation, lung nodule segmentation in the CT images, and left ventricular MRI image segmentation. Experiments demonstrate that Half-UNet has similar segmentation accuracy compared U-Net and its variants, while the parameters and floating-point operations are reduced by 98.6 and 81.8%, respectively, compared with U-Net.

## 1. Introduction

Medical image analysis equipment, including magnetic resonance imaging (MRI), computed tomography (CT), and X-ray imaging have become essential devices for clinical diagnosis. As an essential method for medical image analysis, medical image segmentation provides a reliable basis for clinical diagnosis and early diagnosis of diseases by helping doctors make accurate judgments. Traditional medical image segmentation algorithms mainly include manual segmentation (Mudigonda et al., 2000), semi-automatic segmentation (Kilday et al., 1993; Vard et al., 2011), and automatic segmentation (Qi et al., 2012; Lu et al., 2015). These algorithms rely heavily on human prior knowledge, and have insufficient generalization ability, making it difficult to achieve satisfactory results. Then, deep learning methods drove progress in the field of biomedical image segmentation (Zhang et al., 2015). The earliest Convolutional Neural Networks (CNN) such as LeNet (LeCun et al., 1998), AlexNet (Krizhevsky et al., 2012), VggNet (Simonyan and Zisserman, 2014), and GoogleNet (Szegedy et al., 2015), were introduced to solve image recognition problems.

In recent years, CNN has achieved pixel-level classification by obtaining the classification information of each pixel to solve the problem of image segmentation (Wolterink et al., 2017). Deep convolutional neural networks have a strong ability to extract a large number of features and are rapidly developing for applications in computer vision. The state-of-the-art models for image segmentation utilize distinctive information from different scales, such as fully convolutional networks (FCN) (Long et al., 2015), U-Net (Ronneberger et al., 2015), SegNet (Badrinarayanan et al., 2017), PSPNet (Zhao et al., 2017), and a series of DeepLab versions (Chen et al., 2017a,b, 2018). Among them, U-Net is widely used in medical image segmentation. The U-shaped architecture uses skip connections to combine the high-level semantic feature maps from the decoder and corresponding low-level detailed feature maps from the encoder. A common belief about U-Net is that its success depends on the U-shaped structure, and many U-Net-based models have been proposed. Kerfoot et al. (2018) used a U-Net convolutional neural network architecture built from residual units (He et al., 2016) to segment the left ventricle. Using dense convolutions (Huang et al., 2017) in the U-Net architecture, Li et al. (2018) proposed H-DenseUNet for liver and liver tumor segmentation. UNet++, proposed by Zhou et al. (2018), introduced nested and dense skip connections to reduce the semantic gap between the encoder and decoder. Although reasonable performance can be achieved, the nested network structure is too complex and cannot examine enough information from the full scale. Weng et al. (2019) proposed NAS-UNet, using three types of primitive operation sets and search space to automatically find two cell architectures, DownSC and UpSC, for medical image segmentation, which attains better performance and uses much fewer parameters (about 0.8 M) than standard U-Net. UNet3+, proposed by Huang et al. (2020), uses comprehensive skip connections to aggregate feature maps of all scales at each feature fusion, making more complete use of full-scale feature information. Reasonable results can be obtained using UNet3+, but with fewer parameters than U-Net. DC-UNet, proposed by Lou et al. (2021), analyzed the classical U-Net and the recent MultiResUNet (Ibtehaz and Rahman, 2020) architecture, and then designed the Dual-Channel CNN block to provide more effective features with fewer parameters. However, all these networks follow and rely on the U-shaped structure of the U-Net. More importantly, there is still room for further reduction of parameters and floating-point operations (FLOPs).

Considering the limitations, this article analyzes the U-Net architecture. According to our experimental results, the Half-UNet network model is proposed. In summary, the main contributions of this article are as follows: (i) Experiments show that the excellent segmentation performance of U-Net, similar to the feature pyramid network (FPN), comes from the divide-and-conquer strategy in the encoder, rather than feature fusion in the decoder. (ii) A simple and efficient asymmetric architecture, Half-UNet, is proposed, which uses three strategies to reduce network complexity, including the unification of channel numbers, full-scale feature fusion, and a Ghost module. (iii) Three medical image segmentation datasets are used to compare Half-UNet, U-Net, and variants of U-Net. Experiments show that Half-UNet achieves comparable results with U-Net and its variants, with at least 98.6% fewer parameters and 81.8% fewer FLOPs compared with U-Net. (iv) We found the abnormal gap in parameters and FLOPs between U-Net and UNet3+. Through the analysis of the network structures and formulas, the causes of this abnormal phenomenon are clarified, which also reveals the reason for requiring fewer parameters and FLOPs with Half-UNet.

## 2. Experiments and Analysis of U-Net

Although U-Net is widely used, the question of whether the U-shaped symmetric framework is optimal still exists, including which part of the U-shaped structure dominates the experimental results. Recently, Chen et al. (2021) conducted comparative experiments on Multiple-in-Multiple-out (MiMo), Single-in-Multiple-out (SiMo), Multiple-in-Single-out (MiSo), and Single-in-Single-out (SiSo) encoders. The experiments show that the SiMo encoder can almost achieve the same performance as the MiMo encoder (such as FPN; Lin et al., 2017). This result suggests that the benefits of using multi-scale feature fusion are far less than those of the divide-and-conquer strategy. The network of the U-Net architecture is similar to the FPN. The divide-and-conquer strategy is embodied in the encoder of U-Net, which divides the input image into five different scales of feature maps for output to the decoding layer. On the other hand, the feature fusion strategy is embodied in the decoder of U-Net, which transforms five different scales of feature maps from the encoder into a single-scale feature map after running same-scale feature fusion four times. It is still unclear whether the benefits of U-Net mainly come from the divide-and-conquer strategy, similar to the FPN.

**Figure 1:**
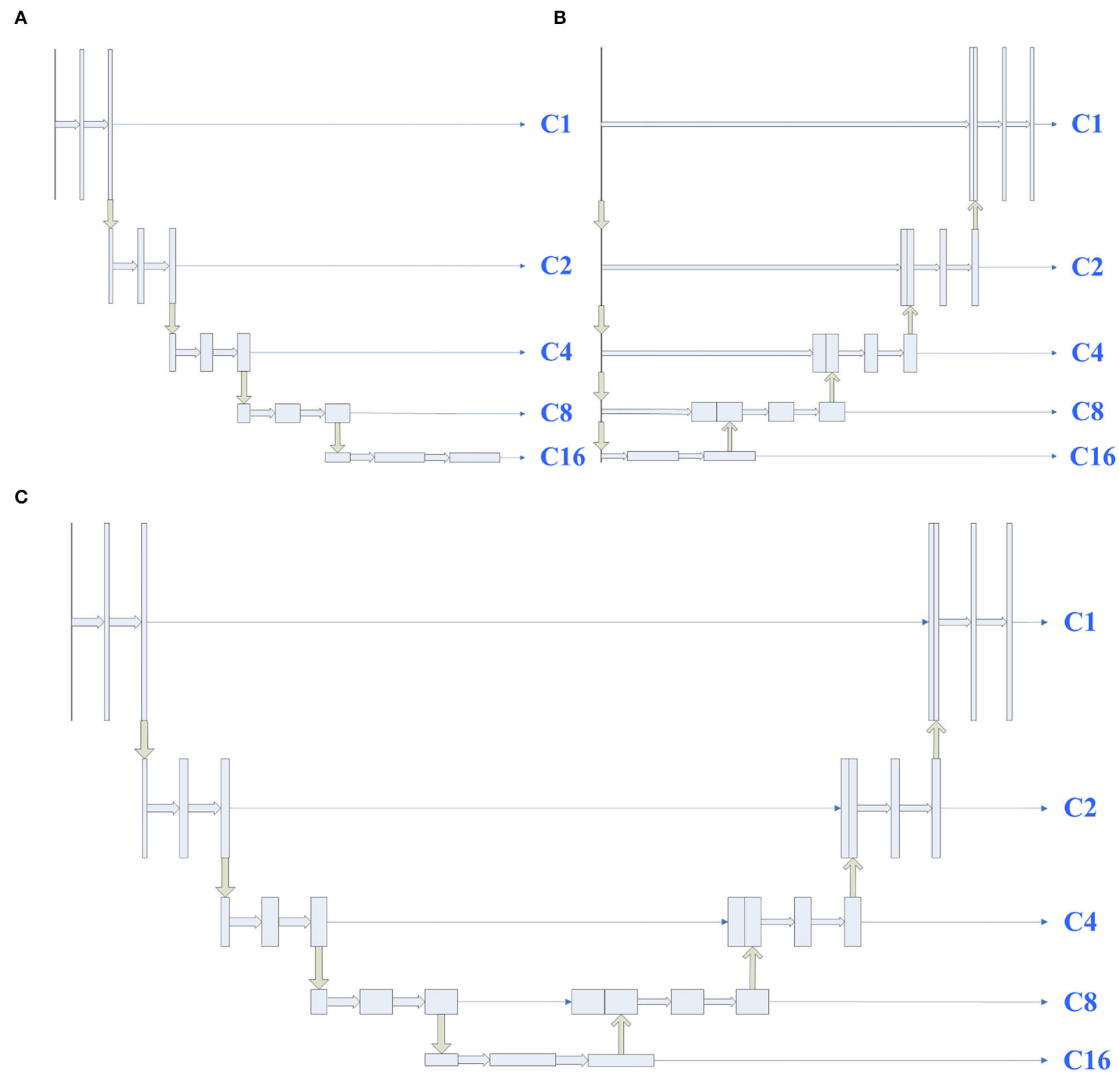
Illustrations of different types of encoders, the structures of encoders **(A–C)** are derived from UNet's encoder, decoder, and full structure, respectively. C1 represents a feature map of the same size as the input map, and C2, C4, C8, and C16 denote output features of the encoder with a downsample rate of {2, 4, 8, 16}. The yellow down (up) arrow represents downsampling (upsampling), and the right thick (thin) arrow represents convolution (copy).

To study the influence of U-Net's encoder and decoder, shown in Figure 1, we considered U-Net's encoder and decoder as encoders. Then, the features from C1 to C16 are aggregated by designing a single decoder, where the structure is the same as full-scale feature aggregation in UNet3+. After that, to prevent the designed decoder from affecting the experimental results, we also used the U-Net complete structure as the encoder in Figure 1C. The experimental results are shown in Table 1. As expected, the encoder (A) can achieve comparable performance with the encoder (C), which demonstrates that the lack of feature fusion in the UNet's decoder has no significant effect on the experimental results. On the other hand, the performance obviously drops in the encoder (B), which shows that the divide-and-conquer strategy in UNet's encoder dominates the experimental results. In summary, the benefits of feature fusion are less significant than the benefits of divide-and-conquer. In other words, if the feature fusion part of U-Net is simplified, then comparable segmentation results can still be obtained.

**Table 1:** Experimental results of different kinds of encoders.

**Network model**	**Mammography**	**Lung nodule**	**Endocardium**	**Epicardium**
Encoder (A)	0.8928	0.8867	0.8901	0.9328
Encoder (B)	0.8744	0.8803	0.7696	0.8158
Encoder (C)	0.8923	0.8878	0.8811	0.9280
U-Net	0.8939	0.8842	0.8797	0.9299

*The dice coefficient was used as the evaluation metric for each case. Encoder (A), (B), and (C) are all connected to general decoder. The structures of the encoder (A), (B), and (C) are shown in Figure 1*.

## 3. Methods

**Figure 2:**
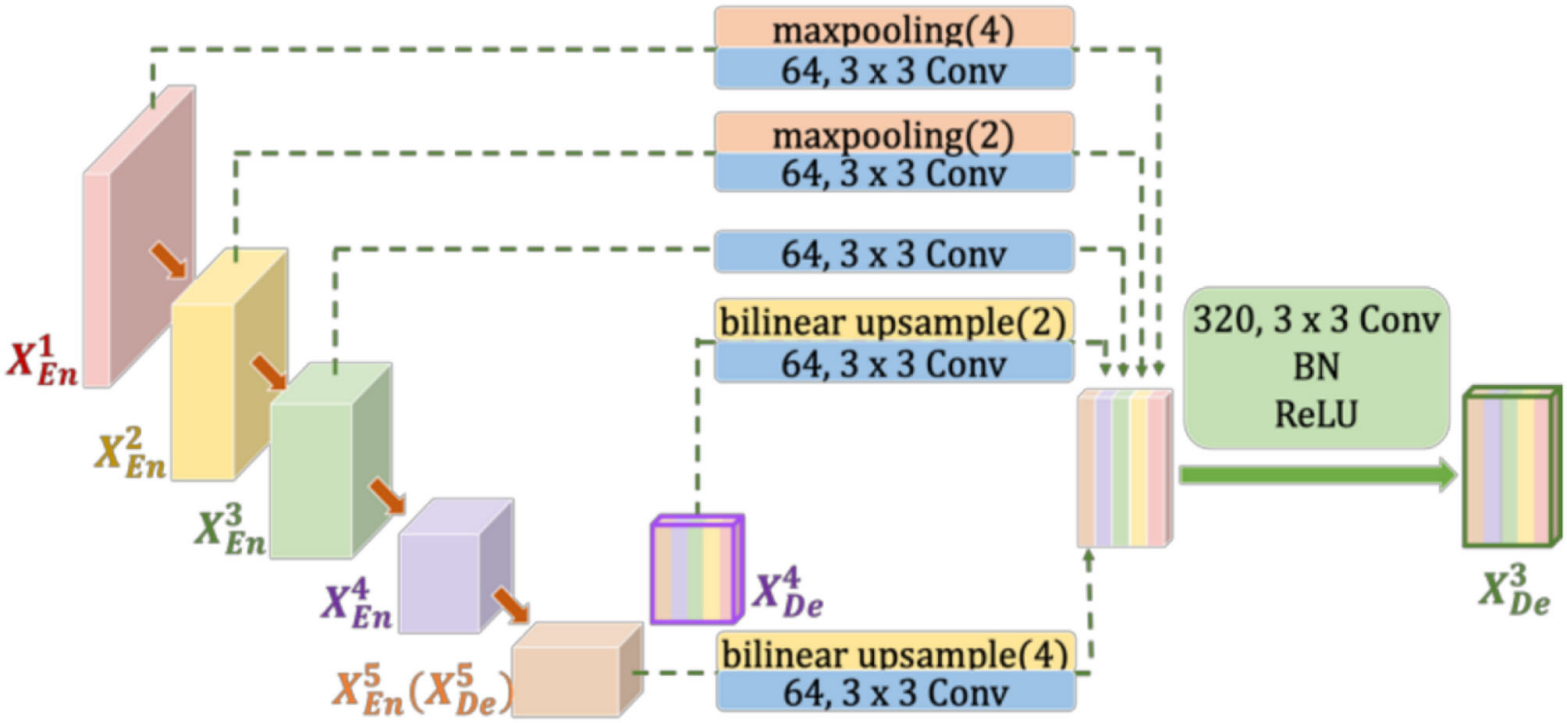
Illustration of how to construct the full-scale aggregated feature map in the third decoder layer of UNet3+. $X_{En}^{1}\left(X_{De}^{1}\right)$ to $X_{En}^{5}\left(X_{De}^{5}\right)$ represent the feature maps of the first to fifth layer encoders (decoders), respectively.

**Figure 3:**
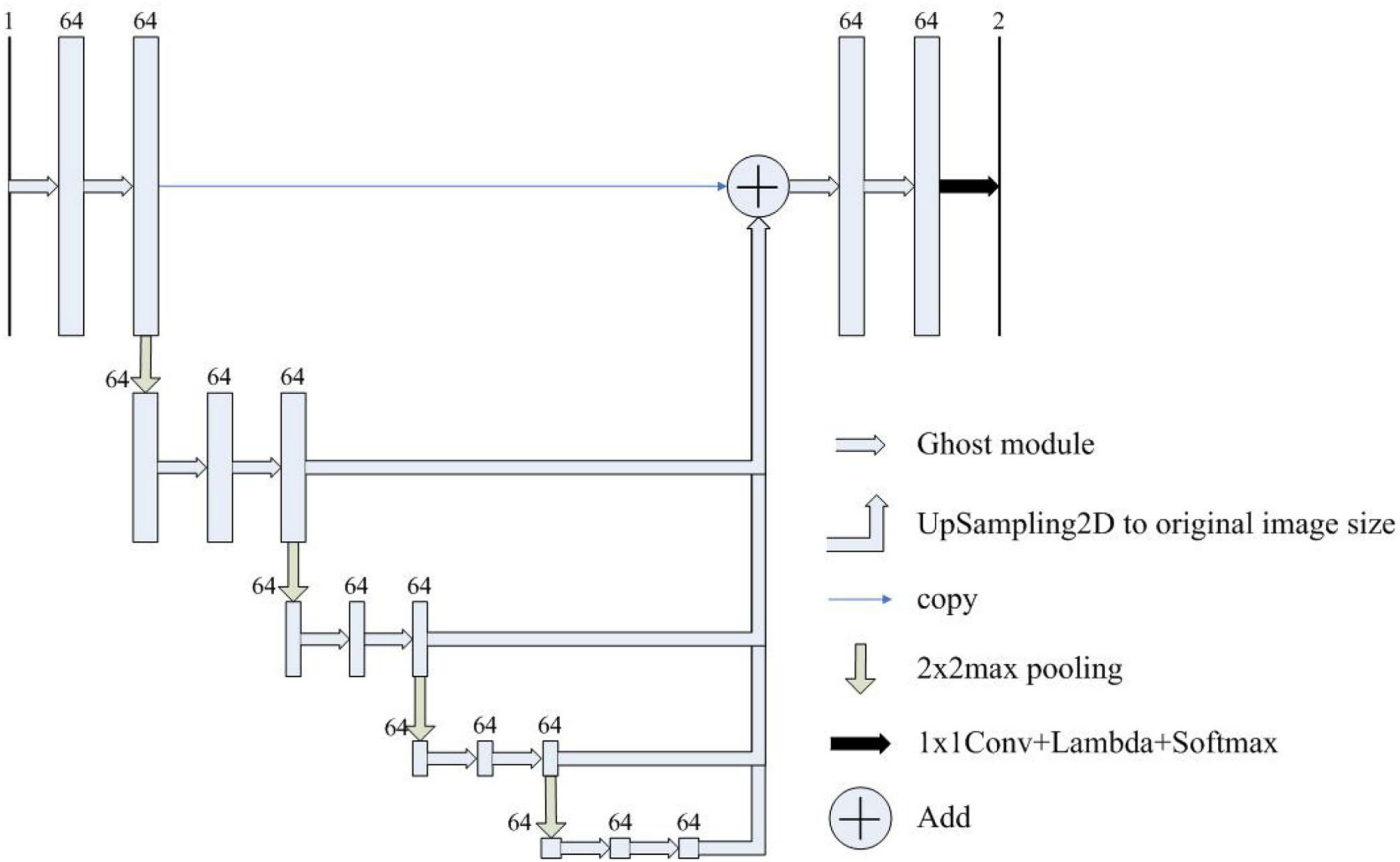
The architecture of Half-UNet. The input image size is detailed in Table 2. The numbers above the rectangles represent the number of feature map channels.

Inspired by the above observations, we concluded that the U-Net's decoder can be simplified to reduce the complexity of the model. For example, the four feature fusions in U-Net can be replaced with the full-scale feature aggregation that is used in UNet3+. However, as shown in Figure 2, the additional 3 × 3 convolutions are added before feature aggregation. Moreover, concatenate operations require more memory overhead and computation. To solve these problems, Half-UNet is proposed, as shown in Figure 3. First, the channel numbers in Half-UNet are unified. This simplifies the network and contributes to the feature fusion of the decoder. Then, to avoid extra parameters and FLOPs required by full-scale feature aggregation, full-scale feature fusion is proposed to replace the four same-scale feature fusions in U-Net. Finally, the Ghost module (Han et al., 2020) is introduced to generate equivalent feature maps at a lower cost.

### 3.1. Unify the Channel Numbers

In each downsampling step of U-Net and UNet3+, the number of feature channels is doubled, which enhances the diversity of feature expression. However, this increases the complexity of the model, especially in UNet3+. As shown in Figure 2, due to the unequal number of channels, 3 × 3 Conv must be added after max pooling (or bilinear upsample) to unify the channel numbers. Furthermore, the added 3 × 3 convolutions increase the required parameters and FLOPs. In Half-UNet, on the other hand, the channel numbers of all feature maps are unified, which reduces the number of filters in the convolution operation and contributes to the feature fusion of the decoder because the decoder does not need to add any additional 3 × 3 convolutions.

### 3.2. Full-Scale Feature Fusion

Both U-Net and UNet3+ use concatenate operations for feature fusion. Concatenate operations are an intuitive feature fusion method, but they also require more memory overhead and computation. ResNet (He et al., 2016) uses the addition operation, another feature fusion method, to simply perform identity mapping and add their outputs to the outputs of the stacked layer. The addition operation does not increase the dimension of describing the image but increases the amount of information under each dimension, which is beneficial for the final image segmentation. More importantly, the addition operation does not require additional parameters or computational complexity.

The proposed full-scale feature fusion combines feature maps from all scales, which can capture fine-grained details and coarse-grained semantics at full scale. As shown in Figure 3, feature maps from different scales are first upsampled to the size of the original image, and then feature fusion is performed through the addition operation.

### 3.3. Ghost Module

**Figure 4:**
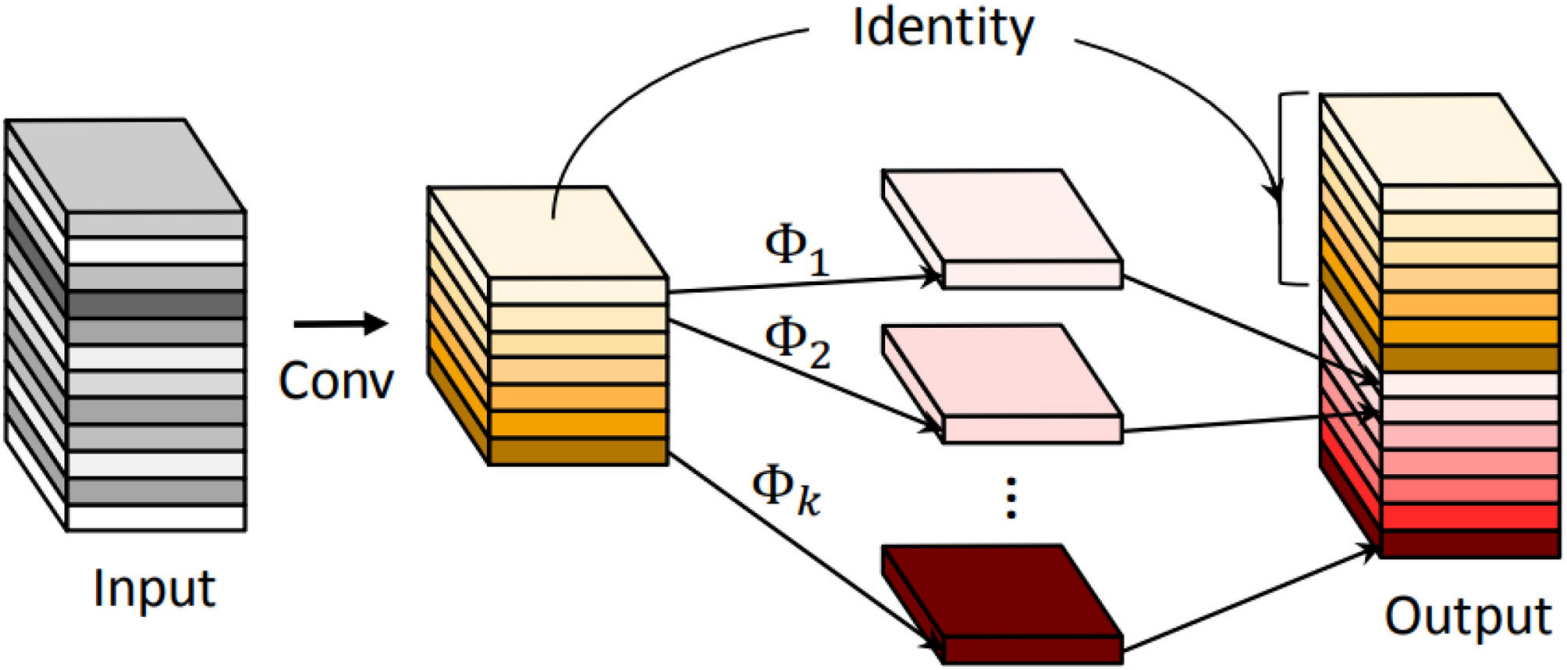
An illustration of the convolutional layer and the introduced Ghost module for outputting the same number of feature maps. Φ represents the cheap operation.

During the convolution procedure, the required parameters and FLOPs can be calculated as


(1) $$\begin{array}{l} Params=\left(K^{2}*C_{in}+1\right)*C_{out} \end{array}$$

(2) $$\begin{array}{l} FLOPs=2*K^{2}*C_{in}*C_{out}*H_{out}*W_{out} \end{array}$$

where K is the kernel size, C_*in*_ (C_*out*_) is the number of input (output) channels, and H_*out*_ (W_*out*_) is the height (width) of the output maps. Han et al. (2020) proposed the Ghost module to generate more feature maps while using cheap operations. An illustration of the Ghost module is shown in Figure 4. During the Ghost module (s = 2, s represents the reciprocal of the proportion of intrinsic feature maps), half of the feature maps are generated by convolution, and the other half are generated by depthwise separable convolution. Finally, the two halves of the feature map are concatenated to form an output of the same dimension as the input. Thus, the parameters and FLOPs can be calculated as


(3) $$\begin{array}{l} Params=\left[K^{2}*\left(C_{in}+1\right)+2\right]*C_{out}/2 \end{array}$$

(4) $$\begin{array}{l} FLOPs=2*K^{2}*\left(C_{in}+1\right)*C_{out}/2*H_{out}*W_{out} \end{array}$$

For example, take a 3 × 3 convolution with an image size of 128 × 128, and both input and output channels are 64 an example. In this case, the required parameters and FLOPs are 36.92 K and 12.08 G. While using the Ghost module, the required params and FLOPs are only 18.78 K and 0.61 G. Therefore, the Ghost module is used in Half-UNet to reduce the required parameters and FLOPs.

## 4. Experiments and Results

### 4.1. Datasets and Data Augmentation

We validate our network model based on three public data sets, as shown in Table 2. There are relatively few images available in mammography and left ventricular MRI data sets. Therefore, we carry out data augmentation on the images of these two training sets: rotate clockwise every 45° for a total of seven times, one horizontal flip and one vertical flip, so that the number of images in the training set is increased by 10 times.

**Table 2:** The medical image segmentation datasets used in our experiments.

**Dataset**	**Images**	**Input size**	**Provider**
Mammography	4,083	128 × 128	DDSM
Lung nodule	4,104	64 × 64	LIDC-IDRI
Left ventricular MRI	5,685	256 × 256	MICCAI 2009

*The “Images” column in the table indicates the number of images after data augmentation*.

The mammography data set comes from The Digital Database for Screening Mammography (DDSM) database of the University of South Florida in the United States. The 483 mammography regions of interest containing masses are sorted and selected, of which 400 images are used as the training set and 83 images are used as the test set.

The lung nodule data set is from the LIDC-IDRI (Armato et al., 2011) public database of lung nodules, which contains 1,018 cases (4,104 images). Because LIDC-IDRI only has detailed contour coordinate information for lung nodules with a diameter ≥3 mm, we select CT images with lung nodules ≥3 mm in diameter. Then, the ground-truth of lung nodules is generated according to the 50% agreement principle. The 50% agreement principle states that two or more out of four doctors consider the pixel area to be a lung nodule and is considered the gold standard for determining lung nodules. Finally, the data are divided into training sets and test sets using a 7:3 ratio.

The left ventricular MRI data set is provided by MICCAI 2009 and contains short-axis images of cardiac MRI scans from multiple cases. There are 45 cases in MICCAI 2009, divided into three groups, and each group contains 15 cases, including 4 with ischemic heart failure, 4 with non-ischemic heart failure, 4 with myocardial hypertrophy, and 3 that are normal. Among them, 30 cases (542 images) are used as the training set and 15 cases (265 images) are used as the test set. All of the left ventricular MRI cases have endocardium, and some of them have epicardium.

### 4.2. Implementation Details

To make a fair comparison, all networks are trained with Adaptive Moment Estimation (Adam) for 60 epochs with an initial learning rate of 0.001. The learning rate is reduced by 2 and 10 at epochs 30 and 50, respectively. Mini-batch of 14 images in mammography and lung nodule datasets. Since the left ventricle MRI images are very large, the mini-batch is only set to 2. The loss function used in each method is Dice loss. The validation ratio is 0.2. Kaiming initialization (He et al., 2015) and L2 regularization are used in Half-UNet by default. All experiments are repeated six times, and the average value is taken as the experimental result.

### 4.3. Evaluation Indicators

In this article, the segmentation performance is evaluated from the Dice coefficient, sensitivity, and specificity. The calculation method of the Dice coefficient is twice the area of the overlapping area between the model prediction result area and the ground-truth, divided by the sum of the two areas. If the Dice coefficient is higher, then the prediction results of the model are more similar to the ground-truth, and the image segmentation results are relatively improved. Let the model prediction result area be P and the ground-truth of breast lumps be M, then the calculation for the Dice coefficient is


(5) $$\begin{array}{l} Dice=\frac{2|P\cap M|}{\left|P|+|M\right|} \end{array}$$

Sensitivity represents the proportion of all positive examples that are correctly predicted and measures the ability of the classifier to identify positive examples. The calculation for sensitivity is


(6) $$\begin{array}{l} Sensitivity=\frac{TP}{P} \end{array}$$

Specificity represents the proportion of all negative examples that are predicted to be correct and measures the classifier's ability to identify negative examples. The calculation for specificity is


(7) $$\begin{array}{l} Specificity=\frac{TN}{N} \end{array}$$

### 4.4. Experimental Results

We compare the proposed Half-UNet with U-Net and variants of U-Net in the task of image segmentation for mammography, lung nodule, endocardium, and epicardium identification. Parameters and FLOPs are used as indicators of the network requirements. The Dice coefficient is used as a measure of network segmentation performance. Table 3 summarizes the quantitative comparison results. For segmentation of mammography and lung nodule images, U-Net and its variants have advantages over Half-UNet. On the other hand, the segmentation by Half-UNet of left ventricular MRI images is improved. Furthermore, we also remove the Ghost modules in Half-UNet, denoted Half-UNet†. As shown in Table 3, Half-UNet† outperforms U-Net and its variants in regard to mammography images and is closer to them than Half-UNet in terms of lung nodule images. However, Half-UNet† performed less well than Half-UNet for left ventricular MRI images. For Half-UNet, the results show that the Ghost module performs well for left ventricular MRI images, and it is less effective for mammography and lung nodule images. In conclusion, Half-UNet (with and without Ghost modules) has similar segmentation accuracy compared with U-Net and its variants, while the parameters and FLOPs are reduced by 98.6 and 81.8%.

**Table 3:** Comparison of U-Net and its variants and the proposed Half-UNet on three datasets.

**Architecture**	**Params**	**FLOPs**	**Mammography Dice**	**Lung nodule Dice**	**Endocardium Dice**	**Epicardium Dice**
U-Net	31.04 M	11×	0.8939	0.8842	0.8797	0.9299
UNet3+	26.97 M	43×	0.8920	0.8864	0.8633	0.9316
DC-UNet	10.07 M	6×	0.8940	0.8855	0.9059	0.9503
Half-UNet*†_u	20.03 M	20×	0.8911	**0.8873**	0.8691	0.8976
Half-UNet*†_d	38.09 M	7×	0.8922	0.8853	0.8926	0.9107
Half-UNet†	0.41 M	2×	**0.8944**	0.8858	0.8794	0.9281
Half-UNet	**0.21 M**	**1×**	0.8892	0.8821	**0.9122**	**0.9555**
			**Sensitivity**	**Sensitivity**	**Sensitivity**	**Sensitivity**
U-Net	31.04 M	11×	0.8745	0.9037	0.8475	0.9097
UNet3+	26.97 M	43×	0.8738	0.9033	0.8345	0.9134
DC-UNet	10.07 M	6×	0.8804	0.9046	0.8906	0.9310
Half-UNet*†_u	20.03 M	20×	0.8725	0.8971	0.8547	0.8877
Half-UNet*†_d	38.09 M	7×	0.8763	0.8916	0.8914	0.9027
Half-UNet†	0.41 M	2×	**0.8875**	0.9131	0.8773	0.9209
Half-UNet	**0.21 M**	**1×**	0.8821	**0.9208**	**0.9029**	**0.9488**
			**Specificity**	**Specificity**	**Specificity**	**Specificity**
U-Net	31.04 M	11×	**0.9942**	0.9941	0.9995	**0.9991**
UNet3+	26.97 M	43×	0.9939	0.9939	**0.9995**	0.9991
DC-UNet	10.07 M	6×	0.9934	0.9945	0.9995	0.9994
Half-UNet*†_u	20.03 M	20×	0.9938	0.9946	0.9994	0.9989
Half-UNet*†_d	38.09 M	7×	0.9933	**0.9949**	0.9993	0.9989
Half-UNet†	0.41 M	2×	0.9926	0.9931	0.9992	0.9989
Half-UNet	**0.21 M**	**1×**	0.9923	0.9925	0.9994	0.9990

The symbol ^†^ means that the Ghost module is not used, and * indicates that the numbers of channels are not unified. The best results are highlighted in bold. “_u” represents feature fusion using the Upsampling2D + 3 × 3 convolution strategy. “_d” represents feature fusion using the deconvolution strategy.

To continue investigating the impact of uniform channel numbers and full-scale feature fusion on experimental results, Half-UNet*†_u and Half-UNet*†_d are designed. Similar to U-Net and its variants, the channel numbers of Half-UNet*†_u and Half-UNet*†_d are doubled after downsampling. Because the channel numbers are different, there are two strategies for feature fusion in the decoder: (1) Upsampling2D + 3 × 3 convolution, which is what Half-UNet*†_u and UNet3+ do; (2) Deconvolution, which is what Half-UNet*†_d and U-Net do. As shown in Table 3, Half-UNet*†_u and Half-UNet*†_d increase the required FLOPs and parameters, respectively, compared with Half-UNet†, but the segmentation abilities have not been significantly improved. The strategy of doubling the number of channels after downsampling increases the channel numbers for high-level semantic features. However, for medical image segmentation, high-level semantics and low-level semantic features are both important. This unfair way of adding features will not bring significant performance improvement to the network, but it will significantly increase the complexity of the model, which is not cost-effective.

Compared with U-Net, Half-UNet*†_u and Half-UNet*†_d only simplify the feature fusion part, while there is no obvious difference in their segmentation ability. This once again shows that U-Net's effective segmentation ability mainly comes from the divide-and-conquer strategy, rather than the feature fusion. The divide-and-conquer strategy divides the complex segmentation problem into multiple sub-problems at the image scale. Ultimately, a more efficient strategy for dividing sub-problems will provide improved segmentation results.

### 4.5. Qualitative Comparison

**Figure 5:**
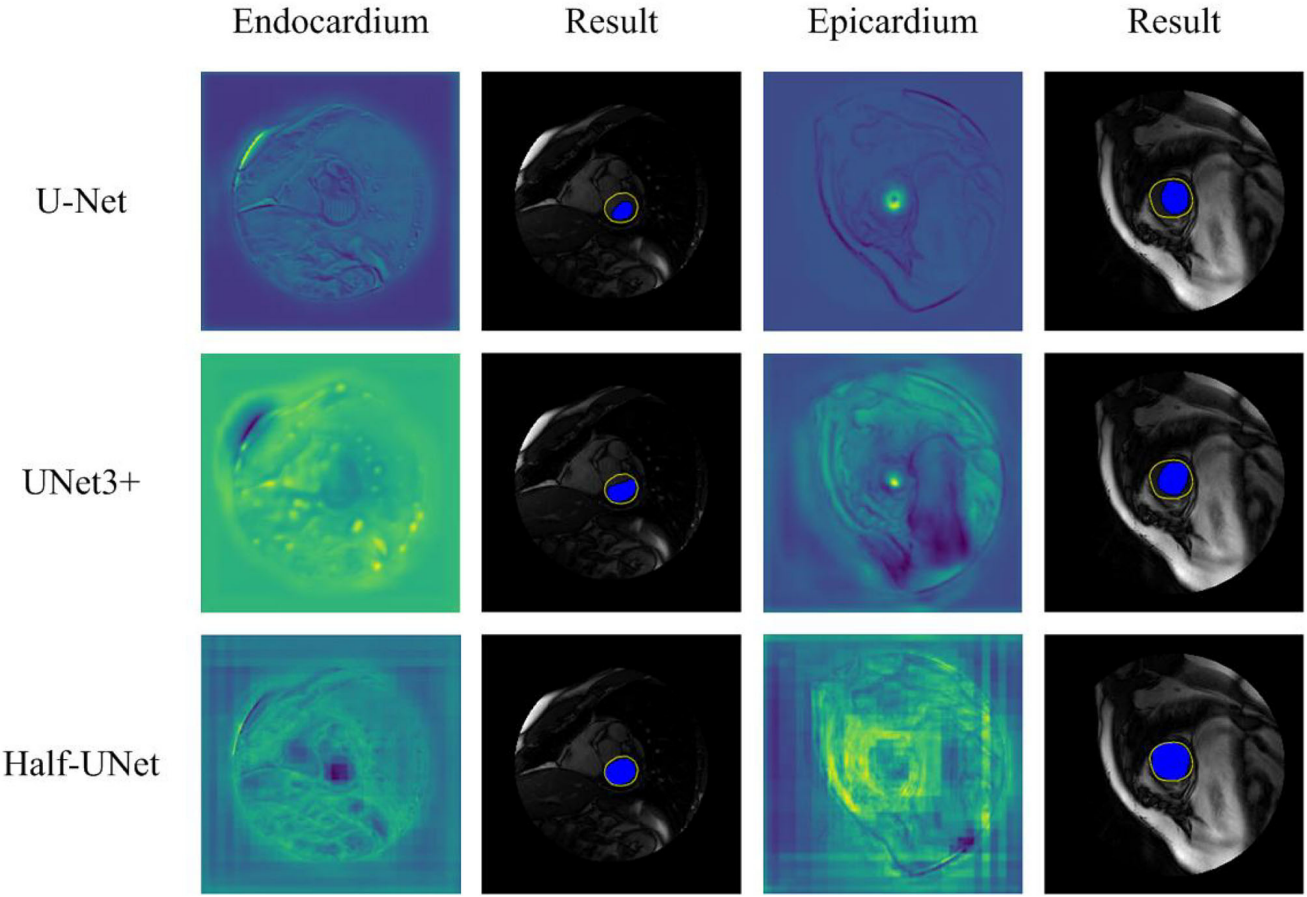
Qualitative comparison between Half-UNet, U-Net, and UNet3+ in left ventricular MRI. Endocardium and Epicardium columns show feature maps. Result columns show final segmentation results, in which the yellow hollow area represents the ground-truth, and the blue solid area represents the automatic segmentation result.

Figure 5 shows a qualitative comparison of the segmentation ability of Half-UNet, U-Net, and UNet3+ for left ventricular MRI images. The structure of the three networks is different in their feature fusion part, so feature maps of the convolutional layer after their last feature fusion are used as endocardium and epicardium columns. In the endocardium column, the ground-truth regions of the feature map of Half-UNet are more prominent, which is completely covered by the black area. In the epicardium column, the feature maps of U-Net and UNet3+ are more prominent in the ground-truth center area but are not complete enough, and the contours are not obvious. In contrast, the ground-truth contour of the Half-UNet's feature map is more obvious and complete. Accordingly, Half-UNet can segment endocardial and epicardial boundaries more completely.

## 5. Discussion

Our results show that the segmentation efficiency of Half-UNet is higher than that of U-Net and its variants. Compared with the U-shaped structural model, the Half-U-shaped model has higher efficiency and similar segmentation ability. Another notable result is that U-Net involves more parameters than UNet3+ but requires fewer FLOPs.

**Figure 6:**
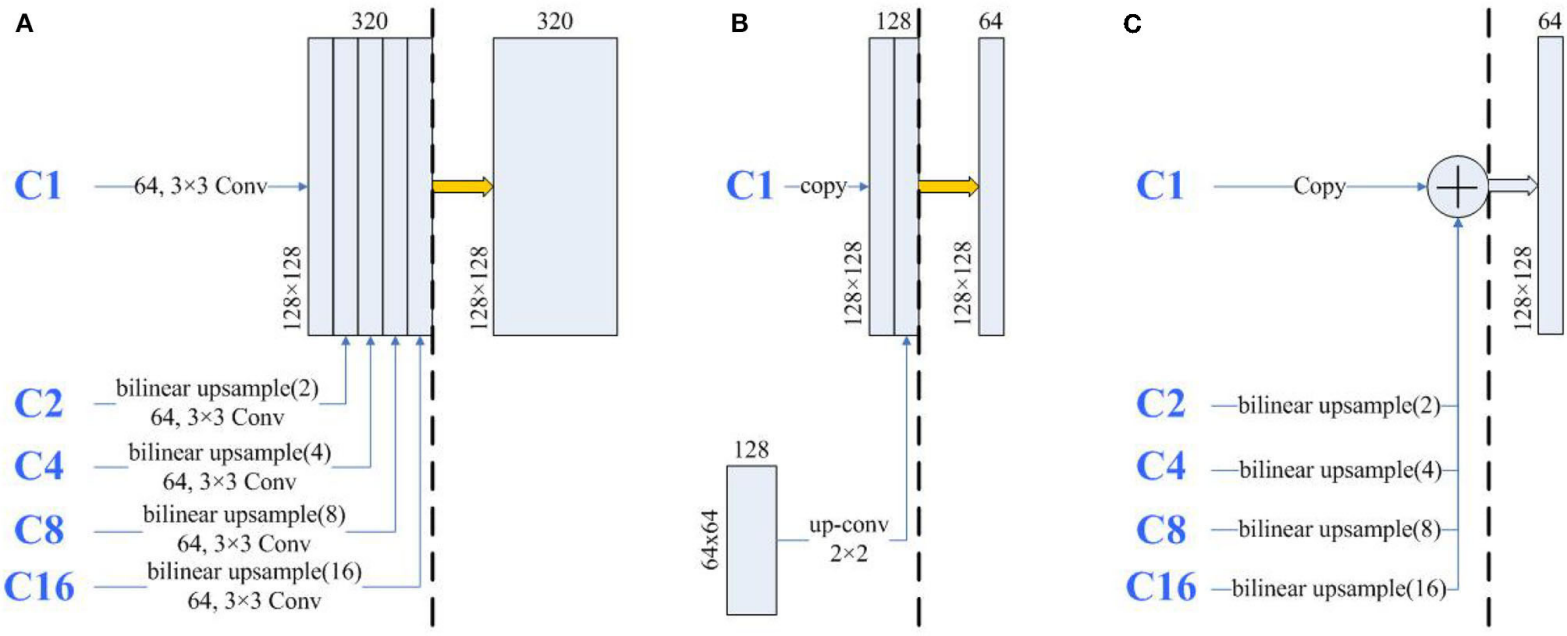
The architecture of the same part of UNet3+ and U-Net. The thick yellow arrows represent 3×3 convolutions and the thick blue arrows represent Ghost modules. The three sub-networks are divided into left and right parts by black dashed lines. **(A)** A part of UNet3+, **(B)** A part of U-Net, and **(C)** A part of-Half-UNet.

To further analyze the high segmentation efficiency of Half-UNet, we intercept the last feature fusion structure of UNet3+, U-Net, and Half-UNet as sub-networks. As shown in Figure 6, each sub-network is divided into the feature fusion part (left part) and convolution part (right part). We use 128 × 128 images as an example, and the parameters and FLOPs of the sub-networks are shown in Table 4. In the left part of the Half-UNet sub-network, since bilinear upsampling and addition are both linear operations, almost no parameters and computation are generated. Half-UNet fuses the feature maps of C1–C16 with the lowest cost and simplifies the feature fusion part. In the right part of the Half-UNet sub-network, due to the lower number of input channels (only 64) and the use of the Ghost module, the cost of convolution is significantly smaller than in other structures.

**Table 4:** In the analysis table of the three sub-networks in Figure 6.

**Sub-network**	**Params**	**FLOPs**	**Sub-network**	**Params**	**FLOPs**
UNet3+_left	1.18 M	38.66 G	UNet3+_right	0.92 M	30.20 G
U-Net_left	0.03 M	0.27 G	U-Net_right	0.07 M	2.42 G
Half-UNet_left	0.00 M	0.00 G	Half-UNet_right	0.02 M	0.61 G

“*_left” and “_right” represent the left and right parts of the black dotted line in Figure 6, respectively*.

As reported in Table 3, U-Net uses more parameters than UNet3+ but uses fewer FLOPs. The first reason is that U-Net has more channels than UNet3+. More channels require more parameters, which can be concluded from Equation (1). The second reason is that U-Net has fewer large-size feature maps than UNet3+. As shown in Figure 6, in UNet3+, C2–C16 is first upsampled to 128 × 128 for a total of 1,984 large-size input feature maps. Moreover, the subsequent channel numbers are more than in U-Net. It can be inferred from Equation (2) that these convolutions in large-size feature maps also require large FLOPs. Similar to U-Net and UNet3+, Half-UNet*†_d has more parameters than Half-UNet*†_u while having fewer FLOPs. The difference is that Half-UNet*†_d has more parameters due to the large size of the deconvolution kernel. Since the deconvolution input images are small, the combined equation with the deconvolution FLOPs is


(8) $$\begin{array}{l} FLOPs=2*K^{2}*C_{in}*C_{out}*H_{in}*W_{in} \end{array}$$

such that Half-UNet*†_d has fewer FLOPs than Half-UNet*†_u. In contrast, Half-UNet does not have an excessive number of channels or large size feature maps or uses deconvolution for upsampling. Half-UNet avoids the problems of the above three networks, significantly reducing the required parameters and FLOPs.

For small targets, such as those found in mammography and lung images, the strategy of obtaining more feature maps by cheap operation does not work well. The result is significantly improved after adding the number of convolution channels, like in Half-UNet*†_u. This suggests that such targets need more spatial features to be effectively segmented. Improving the feature diversity of convolution at a low cost may be a promising direction for future research.

## 6. Conclusion

In this study, we show that the success of U-Net in medical image segmentation is mainly due to its divide-and-conquer solution, rather than feature fusion. Based on this conclusion, Half-UNet is proposed, which mainly simplifies the feature fusion part. Half-UNet simplifies the network complexity by unifying the channel numbers, using full-scale feature fusion, and utilizing Ghost modules. The usefulness of Half-UNet is demonstrated by making fair comparisons with U-Net and its variants. Experimental results show that the proposed Half-UNet obtained results comparable with U-Net and its variants in terms of segmentation performance, while the network complexity is reduced. Finally, by analyzing the gap between parameters and FLOPs in U-Net and UNet3+, the reasons for reduced parameters and FLOPs in Half-UNet are clarified.

## Data Availability Statement

Publicly available datasets were analyzed in this study. These datasets can be found at: http://www.eng.usf.edu/cvprg/Mammography/Database.html; https://wiki.cancerimagingarchive.net/display/Public/LIDC-IDRI; http://sourceforge.net/projects/cardiac-mr/.

## Author Contributions

HL contributed to the conception and design of the study, analyzed the data, and wrote the first draft of the manuscript. SX contributed to dataset collection and comprehensive guidance. HL, YS, and JT contributed to the experiment of the study. All authors contributed to manuscript revision, read, and approved the submitted version.

## Funding

This study was supported in part by the Fundamental Research Funds for the Central Universities, South-Central MinZu University (CZY22015).

## Conflict of Interest

The authors declare that the research was conducted in the absence of any commercial or financial relationships that could be construed as a potential conflict of interest.

## Publisher's Note

All claims expressed in this article are solely those of the authors and do not necessarily represent those of their affiliated organizations, or those of the publisher, the editors and the reviewers. Any product that may be evaluated in this article, or claim that may be made by its manufacturer, is not guaranteed or endorsed by the publisher.
